# Comprehensive Behavioral Testing in the R6/2 Mouse Model of Huntington's Disease Shows No Benefit from CoQ10 or Minocycline

**DOI:** 10.1371/journal.pone.0009793

**Published:** 2010-03-22

**Authors:** Liliana B. Menalled, Monica Patry, Natalie Ragland, Phillip A. S. Lowden, Jennifer Goodman, Jennie Minnich, Benjamin Zahasky, Larry Park, Janet Leeds, David Howland, Ethan Signer, Allan J. Tobin, Daniela Brunner

**Affiliations:** 1 PsychoGenics Inc., Tarrytown, New York, United States of America; 2 School of Biomedical and Chemical Sciences, Birkbeck College, London, United Kingdom; 3 CHDI Management/CHDI Foundation, Los Angeles, California, United States of America; 4 CHDI Management/CHDI Foundation, Princeton, New Jersey, United States of America; 5 CHDI Management/CHDI Foundation, New York, New York, United States of America; 6 Department of Psychiatry, New York State Psychiatric Institute (NYSPI)/Columbia University, New York, New York, United States of America; Emory University, United States of America

## Abstract

Previous studies of the effects of coenzyme Q10 and minocycline on mouse models of Huntington's disease have produced conflicting results regarding their efficacy in behavioral tests. Using our recently published best practices for husbandry and testing for mouse models of Huntington's disease, we report that neither coenzyme Q10 nor minocycline had significant beneficial effects on measures of motor function, general health (open field, rotarod, grip strength, rearing-climbing, body weight and survival) in the R6/2 mouse model. The higher doses of minocycline, on the contrary, reduced survival. We were thus unable to confirm the previously reported benefits for these two drugs, and we discuss potential reasons for these discrepancies, such as the effects of husbandry and nutrition.

## Introduction

Since the identification of the mutation underlying Huntington's disease (HD) much progress has been made in uncovering its complex molecular pathophysiology [1], [2]. However, despite the apparent simplicity of the mutation, a large number of cellular pathways, and molecular targets, have been implicated in the disease pathology [3]. As HD researchers identify more candidate targets and potentially therapeutic compounds, prioritizing projects to move towards clinical trials becomes even more daunting [4].

Until now, HD clinical trials have identified agents that provide symptomatic relief, including selective serotonin reuptake inhibitors for depressive symptoms, benzodiazepines for anxiety, atypical antipsychotics for psychotic symptoms, and, most recently, tetrabenazine for chorea (see review in [5], [6].

Several clinical trials have also evaluated compounds for their ability to alter disease progression, including riluzole, ethyl-eicosapentanoic acid, creatine, coenzyme Q10 (CoQ10), lamotrigine, amantadine and memantine. These compounds show some promise in reducing chorea and motor symptoms. In terms of treatments altering disease progression, several compounds including riluzole, ethyl-eicosapentanoic acid, creatine, CoQ10, lamotrigine, amantadine and memantine have been studied in clinical trials and also shown some relief (see reviews in [5], [7].

CoQ10 is an endogenous substance present in the mitochondrial membrane that acts as an electron acceptor to help generate energy by shuttling electrons during oxidative phosphorylation. It also acts as an antioxidant in both mitochondrial and lipid membranes [8]. While CoQ10 has poor oral bioavailability and brain penetration in both rodents and humans [9], several studies showed significantly increased levels of CoQ10 in plasma and brain following oral supplementation [10], [11], [12]. In various studies using different HD rodent models, CoQ10 extended survival and improved motor behavior, gross brain morphology, aggregate load, ATP levels and indicators of oxidative damage [11], [13], [14], [15], [16], [17], [18]. Beneficial effects have also been reported for other neurodegenerative models, such as the MPTP model of Parkinson's disorder [19].

Minocycline is a second-generation tetracycline antibiotic, which readily crosses the blood-brain barrier [20]. Minocycline has been reported to have effects on caspase activation, neuroinflammation, glutamate excitotoxicity, free radical-induced toxicity and aggregation, all processes implicated in HD pathophysiology [21], [22], [23], [24], [25], [26]. Using behavioral endpoints, two studies reported improvement in rotarod performance and survival in R6/2 mice [15], [25] although others reported either negative or lack of effects in R6/2 [26], no effects in the N171-82Q model [27] and toxicity in the 3-nitroproprionic acid HD model [28].

Given the disparity of results concerning minocycline in the preclinical realm, and the interest in both these compounds in the clinical arena, we chose to reassess their preclinical effects in HD using the R6/2 model. This widely used mouse model recapitulates many of the key features of human HD. In this paper we apply our recently published best practices for husbandry and testing [29] to assess the putative therapeutic effects of CoQ10 and minocycline in R6/2. Our results suggest that neither compound has a robust therapeutic effect in this HD model and that, moreover, minocycline can produce negative effects.

## Materials and Methods

### Subjects

Animal care was in accordance with the *United States Public Health Service Guide for the Care and Use of Laboratory Animals*, and procedures were approved by the Institutional Animal Care and Use Committee at Psychogenics Inc.. R6/2 transgenic mice expressing the N-terminal region of a mutant human huntingtin gene [30] and wild type (WT) littermates were generated by crossing ovarian-transplanted females (from R6/2 CBAxC57Bl/6 female donors) with CBAxC57Bl/6 F1 WT males. Genotype was determined at 15 days of age by PCR of tail snips [31]. Laragen Inc. (USA) measured CAG repeat lengths. All CAG repeat numbers reported here are those determined directly by Genemapper software. Mice were weaned at approximately 3 weeks of age and behavioral tests were performed at 4, 6, 8 and 12 weeks of age, unless otherwise specified.

### Husbandry

Mice were group-housed (4–5/cage) in shoebox cages with wood shavings and a filter top. The environment was enriched with a play tunnel, shredded paper, and a plastic bone. Breeder animals also received cotton nestlets and igloos, instead of play tunnels. Food and water were available *ad libitum*. R6/2 and corresponding WT mice in the minocycline studies received wet powdered food placed inside a cup on the floor of the cage; this additional food was replaced fresh daily and was provided starting at the time of weaning. All mice in the CoQ10 studies received CoQ10-supplemented pelleted food on the floor of the cage. To ensure that calculated doses were obtained, these mice did not received additional wet mush. Temperature (68–76°C), humidity (30–70%) and the light-dark cycle (6:00–18:00 EST) were controlled and monitored daily. Litters of three or less mice were not used. Mice from multiple litters (at least three) were used for each treatment group (n = 20–40; equally divided between genders). A semi-randomized selection process was performed in which mice were grouped matching for body weight and CAG repeat length (mutants only). There were thus, no statistical differences between mutant mice assigned to the different groups within each study. Animals that presented abnormalities such as hydrocephaly, failure to thrive, missing limbs and/or were abnormally small (runts) were excluded from the experimental groups. Body weights were recorded weekly (with the exception of the study with CoQ10 at 0.2%, in which measures were taken biweekly during testing weeks and the study with minocycline 5 mg/kg i.p. when measures were taken three times a week). Cages and animals were carefully manipulated to avoid excess stress or stimulation that may trigger seizures.

### Experimental Procedures

Animals were assigned to the different treatment groups in a semi-randomized fashion, which ensured that there were no significant differences in body weight before the beginning of the testing or of CAG repeat lengths amongst the different treatment groups. The behavioral assays listed below were performed at 4, 6, 8, 10 and 12 weeks of age, unless otherwise noted. Researchers were blind to treatment during experiment testing and data collection. A single investigator conducted each behavioral test. Behavioral testing was always conducted 1 h after lights on and 1 h before lights off.

#### Survival

All mouse cages were examined daily in order to determine lifespan. Mice were noted as dead when they no longer had a heartbeat. Mice were euthanized if found moribund, as defined by lack of movement even after prodding, and/or lying on side and lack of righting reflex.

#### Rotarod

Mice were tested over three consecutive days at 4, 6, 8 and 12 weeks of age using an accelerating rotarod assay (Menalled et al., 2009). Each daily session started in the morning hours, between 9–11 am, and included four trials. The first trial was a training trial of 5 min at 4 RPM on the rotarod apparatus (AccuScan, OH). One hour later, the animals were tested for three consecutive accelerating trials of 5 min with the speed changing from 4 to 40 RPM and an inter-trial interval of at least 30 min. The latency to fall from the rod was recorded. Mice remaining on the rod for more than 300 s were removed and their time scored as 300 s. In less than 1% of the trials, a fecal bolus dropping broke the infrared (IR) beam and terminated the trial prematurely. On these occasions, the trial was restarted for that particular animal and the time to fall was recalculated; these data were removed for analysis for that time point only. To minimize experimenter variability, a single individual performed all tests.

#### Open field

This test was performed during the light phase of the light cycle at 4, 6, 8 and 12 weeks of age. Activity chambers (Med Associates Inc, St Albans, VT; 27×27×20.3 cm) were equipped with infrared beams. Mice were placed in the center of the chamber and their behavior was recorded for 30 min. Quantitative analyses were performed on the following three dependent measures: total locomotion, locomotion in the center of the open field, and rearing rate in the center.

#### Rearing-climbing

To test rearing and climbing abilities, a metal-wire mesh pencil holder was inverted over mice placed on a surface. Climbing was recorded in 5 min-testing sessions that were videotaped for analysis.

#### Grip strength

This test was performed at 4, 6, 8, 10 and 12 weeks of age. However, only data corresponding to the 12-week time point are reported because no deficiencies in grip strength due to genotype were detected prior to this age. To measure forelimb grip strength, a mouse was held by the tail and lowered towards the apparatus (Ugo Basile, Italy) until it grabbed a handle with both front paws. Mice were gently pulled back until they released their grip from the handle. Each session consisted of five consecutive trials. The equipment automatically measures the grams of force required to pry the mouse from the handle.

Animals receiving CoQ10 were also evaluated in the prepulse inhibition of startle test. (No drug effects found. Data not shown.)

### Treatments

Treatments were started at 4 weeks of age ±3 days to maximize the opportunity to detect any treatment effects. Drugs sharing the same vehicle or control treatment were run together. Groups of 10–21 animals per gender per genotype were used in each treatment group.

#### Experiment 1

*CoQ10 0.2%*: Thirty-eight to 40 mice per genotype (19–20 per gender) were used in each treatment group. The mean CAG repeat length for untreated animals was 125.58±1.00 and for treated animals 125.85±0.95. Animals were placed on either an unsupplemented diet or a diet supplemented with 0.2% CoQ10 (Health Weight Products Inc.; Clackmas, Oregon), corresponding to a daily dose of 400 mg/kg assuming a 20 g mouse eats 4 g per day. Both diets were delivered in the form of pelleted mouse chow (Purina Test Diets, Richmond, IN) as described elsewhere [13], [15].

#### Experiment 2

*CoQ10 0.6% with gamma-cyclodextrin*: Twenty mice per genotype (10 per gender) were placed on a diet supplemented with 0.6% CoQ10 with gamma-cyclodextrin (Tishcon, Westbury, NY) containing 20% CoQ10 and 80% gamma-cyclodextrin. This corresponds to a daily dose of 1200 mg/kg. Twenty mice per genotype (9–11 per gender) were placed on an unsupplemented diet containing only gamma-cyclodextrin (2.5%). The mean CAG repeat length for unsupplemented diet group was 124±0.74 and for the treated animals 123.1±0.59. Both diets were made into pelleted mouse chow (Purina Test Diets, Richmond, NY).

#### Experiment 3

*Intraperitoneal minocycline (5 mg/kg)*: Sixteen to 22 mice per genotype (8–11 mice per gender) received daily intraperitoneal injections of either saline or minocycline (5 mg/kg, Sigma Aldrich, St. Louis, MO) starting at 6 weeks of age. The mean CAG repeat length of vehicle-treated animals was 108.56±0.67 and of minocycline-treated animals 109.11±0.72. Daily injection volume was 5 ml/kg. Minocycline solutions were prepared daily to ensure compound stability. Dosage and administration were as described elsewhere [25]. To avoid acute effects, all behavioral testing was conducted at least 3 hours after injections.

#### Experiment 4

*Oral minocycline 0.1% and 0.375%*: Twenty two to 24 mice (11–12 per gender) per genotype received wet food supplemented or unsupplemented with minocycline (0.1% or 0.375%; corresponding to 200 and 750 mg/kg per day, respectively, Sigma-Aldrich, St. Louis, MO). The mean CAG repeat length of vehicle-treated animals was 122.91±0.62, of animals treated with 0.1% minocycline 125.17±0.69, and of animals treated with 0.375% minocycline 124.04±0.91. Minocycline supplemented food was prepared daily by mixing powdered minocycline with dry powdered food. Water was added to the mixture at a 1∶1 weight ratio. The unsupplemented control diet was also made daily in a similar manner. The resulting mush was placed in an open plastic container on the floor of each cage. Pelleted dry food was not given to these animals to ensure they received the complete minocycline dose.

#### CoQ10 measurements

Measurements were made by LC/MS/MS analytical methodology. The system consisted of a Phenomenex Polar RP column, 50×2.1 mm id, 4 µm. The Mobile Phase was 100% methanol, isocratic with a flow rate of 400 µL/min and a total run time of 4.0 min. The injection volume was 40 µL. For MS, the system consisted of a PE Sciex API 4000 Triple Quadrupole with a heated nebulizer interface. Temperature was 400°C. Standards and quality control samples were prepared in neat matrix. Stock solutions of CoQ were prepared in ethanol. Standards were prepared by serial dilution in 5∶1, n-propanol∶water at 2500, 1000, 500, 100, 50, 10, 5, 1 and 0.5 ng/ml. Brain samples were homogenized using a Virsonic 100 ultrasonic homogenizer. Each sample was first weighed and then placed into a 5 ml test tube. An appropriate amount of 20∶80, methanol∶water was added to make a 1 g/4 ml mixture. These samples were then homogenized and the final volumes were measured. All samples were then stored at −80°C pending analysis. Plasma and brain samples were prepared by precipitation with n-propanol. To a 1.7 ml centrifuge tube, 50 µl of appropriate sample was added. To each tube, 12.5 µl of a 2 mg/ml solution of 1,4 benzoquinone was added, capped, vortexed, and left to stand for ten minutes. Addition of 1,4 benzoquinone oxidizes all CoQ10 in the sample to the fully oxidized state, allowing for the determination of total CoQ10 in each sample. To each sample, 250 µl of n-propanol was then added. The samples were vortexed briefly, and then centrifuged at 13000 rpm for ten minutes. A 100 µl aliquot of each sample was then transferred to an HPLC vial for analysis.

#### Minocycline measurements

Minocycline levels in brain and plasma were determined as described elsewhere [26]. Briefly, WT and R6/2 mice (n = 3 per gender, per genotype, per treatment) were fed either minocycline-supplemented powdered food or control chow for 3–4 weeks before collecting tissue. Animals were euthanized with CO_2_ and blood was collected from the chest cavity before dissecting the brain. The blood collection tube, containing EDTA, was gently inverted immediately after sample collection. The samples were then placed on ice and spun at 5000 RPM for 15 min at 4°C in a refrigerated centrifuge. The plasma was then pipetted out of the sample collection tube. Brains were dissected and immediately frozen in powdered dry ice. Levels of minocycline were assessed as described by [32]. In brief, brains were homogenized, and centrifuged for 1 hour at 10,000g at 4°C. The supernatant was stored at −80°C for HPLC analysis. Reverse-phase HPLC was performed using a Waters 600E solvent delivery module and a Waters 484 UV detector. Samples were analyzed on a Hypersil H5ODS column (15cm 4.6mm, Hichrom, Berkshire, UK) by isocratic elution. The minocycline concentrations were calculated against a standard curve made by adding in known concentrations of minocycline to brain or plasma samples and extracting [33].

### Statistics

An alpha level of 0.05 was selected for all inferential statistics. The repeated measures analyses were carried out with SAS (SAS Institute Inc.) using Mixed Effect Model (also known as Mixed ANOVA Model). These analyses are based on likelihood estimation, which is more robust to missing values than moment estimation. The models were fitted using the procedure PROC MIXED [34]. Slight variations of those models were tested and the best model based on the AIC/BIC criteria [35] was selected. In the present study, treatment was considered the most important factor in the model. Gender was included as a factor in the model when it shows a significant interaction with treatment. Time bins and age were considered as correlated measures.

Genotypic differences were analyzed separately from treatment and in all cases matched published results showing progressive deficits in the R6/2 mice in all measures shown [36], [37]. Therefore, genotype as a factor was not included in any of the statistical models and only data from the mutant animals were included in the analysis. The treatment effect on the WT animals, when available, was included in the figures and is discussed for comparison purposes only. As differences in PK/PD between genotypes could change the effects of the drugs, we compared the levels of drugs in brain and plasma of treated versus untreated groups and treated mutants versus WT mice. Significant interactions with treatment were analyzed with simple main effect test followed by Fisher LSD where indicated.

For the rearing climbing test, the latency to climb was categorized as climbing or not climbing. Differences between treatments were analyzed with a Chi Square at each age separately. Gender differences were not apparent so data were pooled across genders.

Survival data were analyzed with Kaplan-Meier analysis (with the *p* values derived from the Mantel-Cox Log-rank statistic).

Power analyses showed that, fixing alpha at 0.05, effect size at 30% and sample size at either 20 or 40 mice per group, we obtained power >0.80/0.97 (n = 20/n = 40) for rearing in the open field (≥12 weeks of age), ≥0.89/0.99 for body weight (≥14 weeks of age) and ≥0.93/0.99 for rotarod (≥12 weeks of age), grip strength and locomotion were the less robust measures with power of 0.66/0.90 for grip strength and 0.19/0.29 for locomotion in the open field (≥12 weeks for both parameters). The open field thus should be used to obtain rearing measures, rather than locomotion (although this endpoint is robust at later ages [29]. The climbing behavior in the rearing-climbing test show high power (>.87) at both sample sizes (≥12 weeks of age).

## Results

### Experiment 1

#### CoQ10 at 0.2%

We tested the effects of oral administration of 0.2% CoQ10 on the survival, weight and behavior of R6/2 mice and measured CoQ10 concentrations in plasma. This dose was chosen based on the study by Ferrante and colleagues, in which CoQ10's therapeutic effects in R6/2 mice were first described [13].

**Figure 1:**
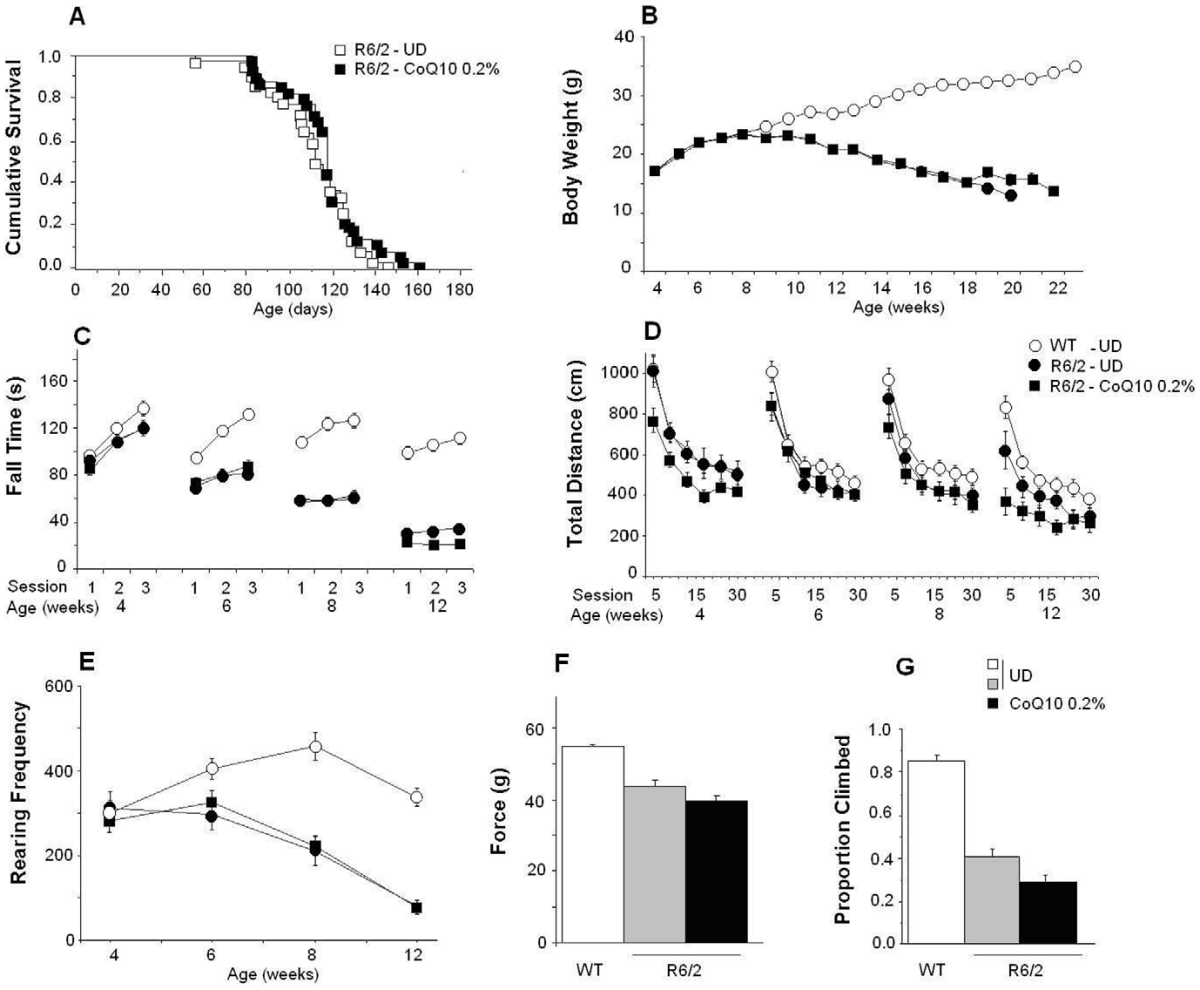
Effect of the administration of 0.2% of CoQ10 in food in R6/2 mice. WT mice received unsupplemented diet (UD) and R6/2 mice received either UD or 0.2% CoQ10. There were no significant treatment effects on survival (A), body weight (B) or rotarod (C: latency to fall). Deleterious effects of 0.2% CoQ10 were observed in the total distance traveled in the open field (D) at 4 and 12 weeks of age, mainly in the first 5 min of the session (treatment×age interaction: F(3,213) = 7.5, p<0.0001; treatment×session time interaction: F(5,375) = 2.5, p<0.04; simple main effects: p<0.05). There were no effects on the rearing frequency in the open field (E); CoQ10 impaired grip strength (F) of female mice at 12 weeks of age (treatment×gender interaction (F(1,76) = 11.3, p<0.002, simple main effects: p<0.001) and decreased the number of mice that climbed in the rearing climbing test (G, p<0.04).

As shown in Fig 1, no beneficial effects of coenzyme Q in the R6/2 mice were detected in survival, body weight, latency to fall from the rotarod, total rearing frequency in the center of the open field, and climbing, in the rearing-climbing test. Non-progressive transient deleterious effects were observed in the total distance traveled in the open field and grip strength.

### Experiment 2

#### CoQ10 at 0.6%

Smith et al. reported that high-dose CoQ10 (0.5%) significantly improved survival and the behavioral phenotype of R6/2 mice, delaying weight loss, motor deficits, and loss of grip strength. In addition, the study suggested that use of the HydroQSorb formulation (Tishcon Corp), is effective at a lower concentration [14]. We administered 0.6% CoQ10 in the HydroQSorb formulation and measured plasma and brain CoQ10 levels, in addition to the behavioral assessment.

**Figure 2:**
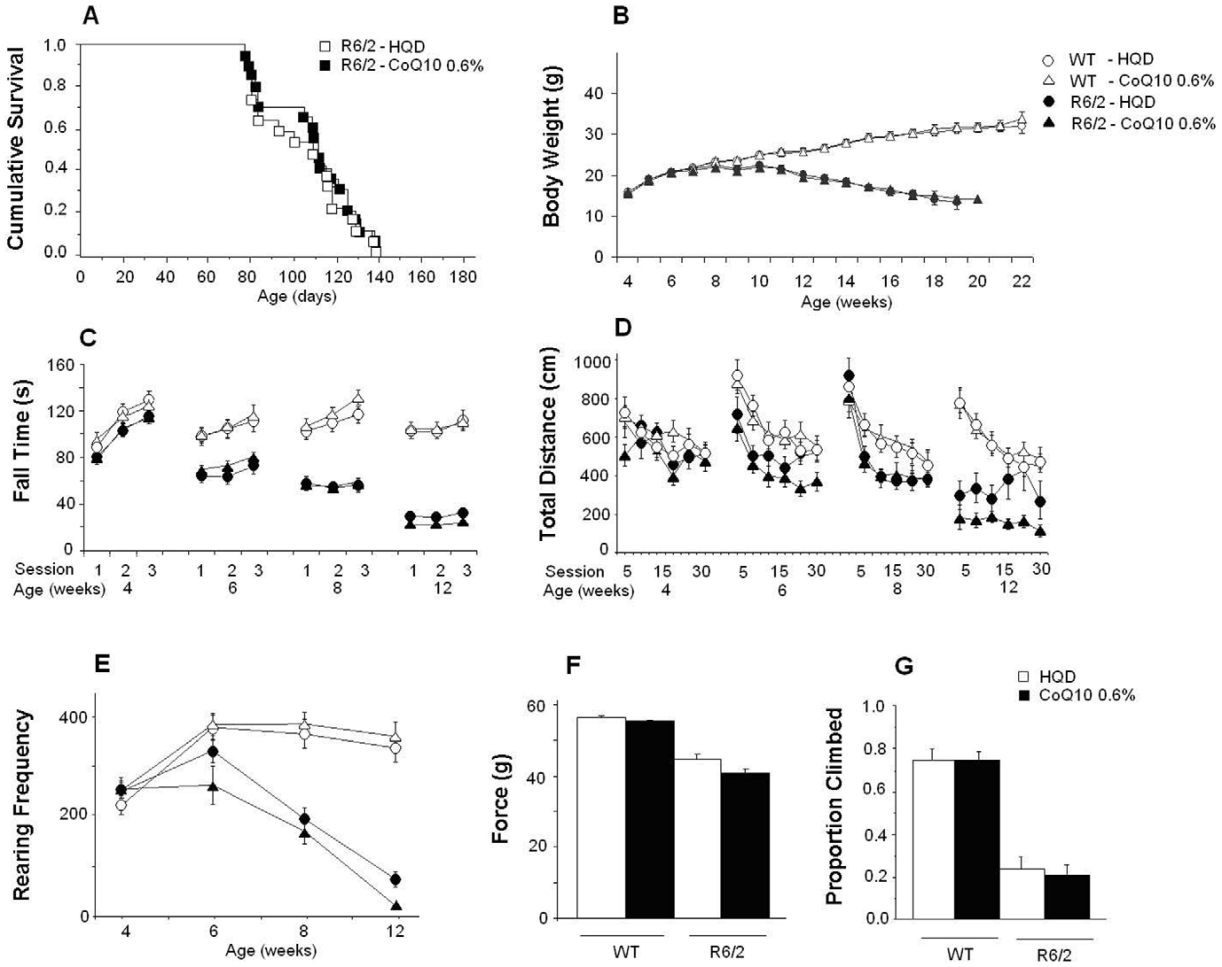
Effect of the administration of 0.6% of CoQ10 in food in R6/2 mice. WT mice and R6/2 mice were fed with a diet with HydroQsorb (HQD) alone or with 0.6% CoQ10 in HQD. There was no treatment effect on survival (A). CoQ10 significantly decreased the body weight (B) of R6/2 males between 6 and 10 weeks of age (treatment×age×gender interaction: F(22,330) = 4.1, p<0.0001; simple main effects: ps<0.05). There were no effects of treatment on the latency to fall from the rotarod (C). In mutant females, CoQ10 diminished locomotor activity in the open field (D). Treated R6/2 females were significantly hypoactive at 4 weeks (0–5 min), 6 weeks (last 10 min), at 12 weeks of age (20 to 25 min; treatment×gender interaction: F(1,35) = 6.7, p<0.02; simple main effects: p<0.007; treatment×age×time interaction: F(30,460) = 3.4, p<0.0001; simple main effects: ps<0.05). R6/2 treated mice reared significantly less in the open field (E) at 6 weeks of age compared with untreated mutant mice (treatment×age interaction: F(3,92) = 3.2, p<0.03; simple main effects: p<0.035). There were no treatment effects on either grip strength (F) or in the rearing climbing test (G). CoQ10 had no effects in WT mice (A–F).

As shown in Fig. 2, CoQ10 did not have beneficial effects on survival, rotarod performance, grip strength performance and climbing although transiently decreased body weight, locomotor activity and rearing in R6/2 mice with no effects in WT.

Plasma levels of CoQ10 were higher in treated than in untreated mice, regardless of the genotype (Table 1). Treated R6/2 mice had higher plasma CoQ10 levels than treated WT mice. Concentrations were higher in females than male mice. We also found a smaller but significant increase in brain CoQ10 levels in treated mice as compared to untreated mice, independent of gender and genotype.

**Table 1:** Mean plasma and brain levels of CoQ10, in 10-week old mice, after 6 w of treatment.

Experiment		Plasma (µg/ml)±SEM				Brain (µg/g)±SEM			
		WT		R6/2		WT		R6/2	
		Female	Male	Female	Male	Female	Male	Female	Male
2	vehicle	0.03±0.01	0.02±0.01	0.04±0.01	0.08±0.01	10.7±0.4	10.7±0.2	11.9±0.7	12.1±0.6
	0.6%	1.3±0.3	0.5±0.8	1.6±0.1	1.0±0.2	13.2+1.6	14.0+1.5	14.2+0.3	13.7+0.5

**Plasma**: Both R6/2 and WT treated mice had higher levels than untreated mice. Treatment main effect: F(1,16) = 173.5, p<0.0001). Treated, but not untreated, mutant mice had higher plasma levels than WT mice (genotype main effect: F(1,16) = 6.1, p<0.026; genotype×treatment interaction: F(1,16) = 6.0, p<0.02, simple main effects: p<0.05). Levels were higher in females than male mice (gender main effect: F(1,16) = 15.7, ps<0.02). **Brain**: CoQ10 levels in animals receiving 0.6% CoQ10 were higher than those of untreated mice, independent of gender and genotype (treatment main effect: F(1,16) = 15.8, p<0.01).

### Experiment 3

#### Minocycline at 5mg/kg

In this experiment, minocycline was injected daily, i.p., at 5 mg/kg, a dose reported to ameliorate the HD phenotype in R6/2 mice by Chen et al. [25].

**Figure 3:**
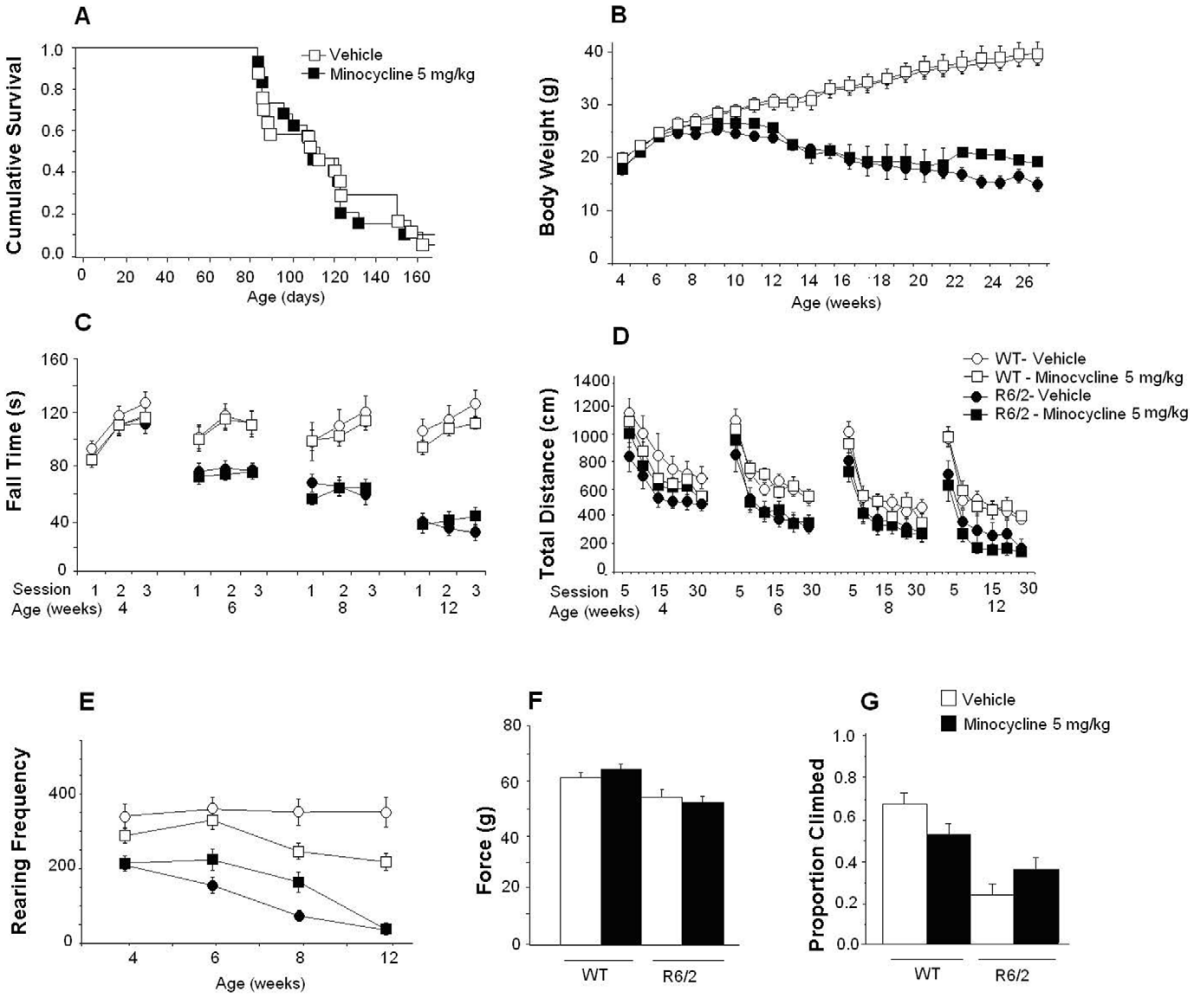
Effect of daily i.p. administration of 5mg/kg of minocycline in R6/2 mice. WT and R6/2 mice received either 5mg/kg of minocycline or its vehicle by intraperitoneal injection. Minocycline had no effects on survival (A) on either R6/2 or WT mice, but transiently increased the body weight (B) of mutant male mice at 8, 10, 11 and 12 weeks of age (treatment×gender×age interaction: F(22,312) = 4.2, p<0.0001; simple main effects: ps<0.05). In the WT group, minocycline did not produce significant effects in body weight. In the rotarod test (C), although there was a triple interaction of treatment, age and day for R6/2 mice, simple main effects did not reveal a consistent pattern (overall effects: treatment×age×day interaction: F(10,143) = 3.2, p<0.001). There were no effects for the WT mice. Minocycline transiently increased locomotion (D) in R6/2 male mice (treatment×gender×age: F(6,87) = 4.5; p<0.0005; simple main effects: ps<0.05 at 4 weeks of age) but decreased locomotion in the male WT mice (treatment×gender×week interaction, F(6,102) = 2.6, p<0.03, simple main effects and post hocs, ps<0.05 at 4 weeks of age). Rearing frequency (E) was transiently decreased by minocycline in female R6/2 mice at 6 weeks, but increased in male R6/2 mice at 6 and 8 weeks of age (treatment×gender interaction: F(1,33) = 11.2, p<0.002; treatment×age interaction: F(3,87) = 6.4, p<0.001; treatment×gender×age interaction: F(6,87) = 5.4, p<0.0001; simple main effects: ps<0.05). Minocycline decreased rearing in WT mice at 8 and 12 weeks of age especially in the male group (treatment×gender interaction, F(1,37) = 9.9, p<0.0005; treatment×age interaction: F(3,102) = 8.9, p<0.0001, simple main effects, ps<0.05). Minocycline did not affect grip strength (F) or rearing (G) in either R6/2 or WT mice although the latter showed a tendency to rear less (p<.07).

As shown in Fig. 3, minocycline did not affect survival, grip strength, rotarod, or climbing although it did transiently increased body weight and locomotor activity in R6/2 mice. Rearing frequency was transiently impaired in female but improved in male R6/2 mice (see legend of figure 3 for statistical details). In the WT group, minocycline did not affect body weight, rotarod or grip strength performance, but transiently impaired locomotion and rearing.

Both WT and R6/2 mice treated with minocycline weeks had detectable concentrations of the compound in both plasma and brain. All minocycline concentrations were higher in wild type mice than in R6/2 mice (Table 2)

**Table 2:** Mean plasma and brain levels of minocycline.

Experiment	Dose	Plasma (uM)±SEM		Brain (uM)±SEM	
		WT	R6/2	WT	R6/2
3	5mg/kg	1.7±0.7	0.8±0.5	0.9±0.1	0.3±0.1
4	0.10% = 1 mg/ml	1.6±0.3	4.5±0.4	2.7±0.4	3.8±0.4
	0.375% = 5 mg/ml	2.4±0.5	7.5±1.2	8.1±0.4	12.5±0.7

**Experiment 3.** Both plasma and brain minocycline levels were higher in WT mice compared to R6/2 mice (Plasma: F(1,19) = 1.4, p<0.01; brain: genotype main effect: F(1,10) = 19.6, p<0.002). **Experiment 4.** Brain and plasma concentrations of minocycline in R6/2 mice were significantly higher than in WT treated mice (Brain: treatment×genotype main effect: F(1,16) = 7.67, p<0.02, simple main effects: p<0.0001; plasma: genotype main effect: F(1,17) = 27.89, p<0.03) in the two 0.375% dosing groups.

### Experiment 4

#### Minocycline at 0.1 and 0.375%

We also tested two oral doses of minocycline based on Smith et al's study [26] in which higher concentrations of plasma and brain minocycline were achieved as compared to the daily i.p. injections of Chen et al's study [25]. Smith and colleagues reported an approximately 10-fold higher concentration of minocycline in brain without ill effects using 1 mg/ml and 5 mg/ml oral minocycline (in the drinking water) compared to a single-dose injection of 5 mg/kg. A single bolus dose is unlikely to result in similar brain exposure compared to that achieved in continuous treatment because of the 2-h plasma half-life of minocycline in mice [38]. Based on these observations, we fed R6/2 and WT mice diets containing 0.1% or 0.375% minocycline assuming that a 20 g mouse eats 4 g of food per day. This is equivalent to doses of 1.3 mg/ml and 5 mg/ml in the drinking water, respectively, assuming that a 20 g mouse drinks 3 ml of water/day.

**Figure 4:**
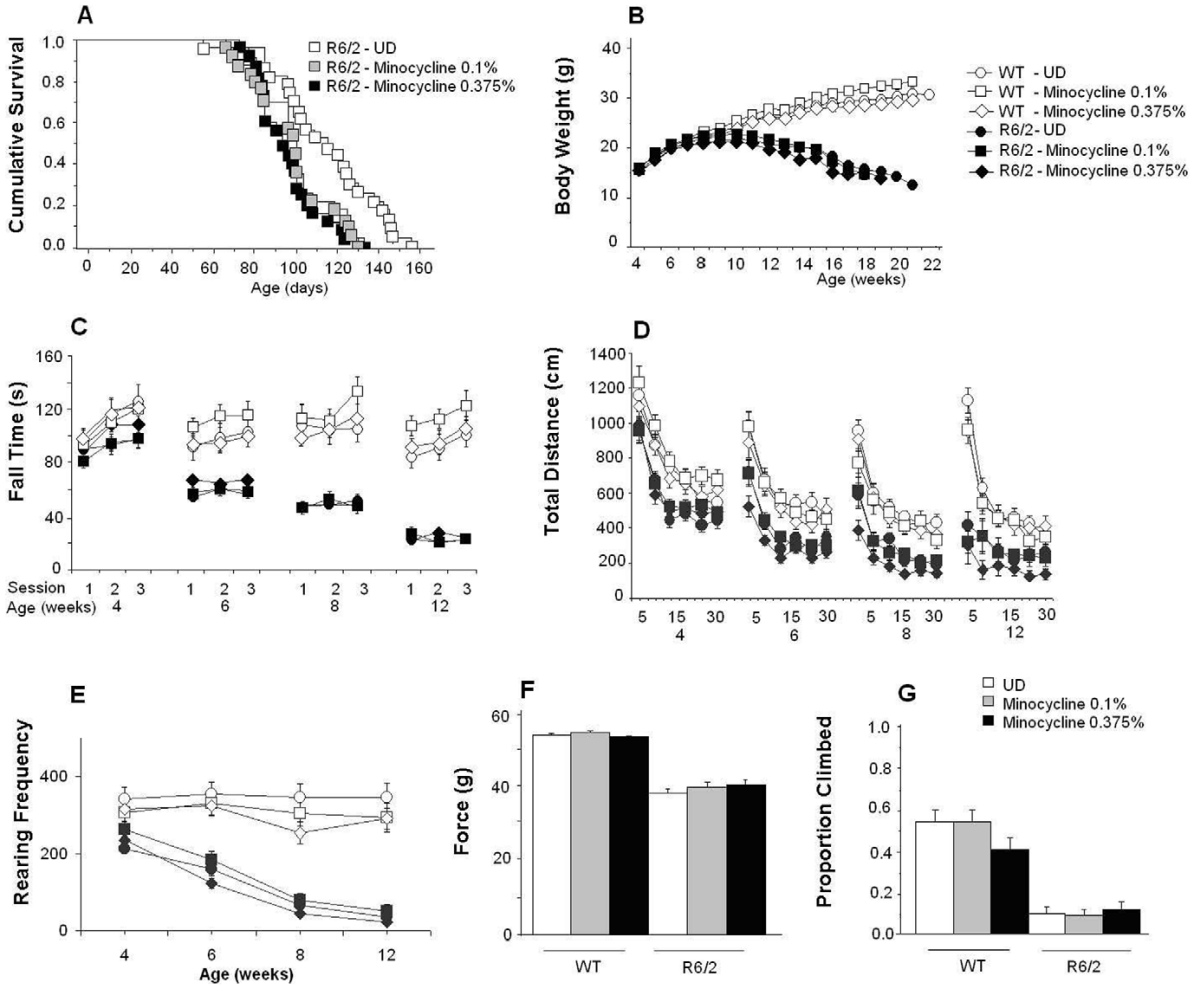
Effect of the administration of 0.1% and 0.375% of minocycline in food in R6/2 mice. WT or R6/2 mice received either a normal diet or a diet supplemented with minocycline (0.1% or 0.375%). Minocycline impaired survival in the R6/2 mice (A, ps<0.01) at both doses. 0.1% minocycline transiently increased body weight (B) in male but not female R6/2 mice, whereas the 0.375% dose decreased body weight in both male and female R6/2 mice (0.1% dose: treatment×age interaction: F(22,620)  =  2.0, p<0.05; simple main effects: ps<0.03 at 7 to 11 weeks of age. 0.375% dose: treatment×age×gender interaction: F(33,620) = 2.9, p<0.0001; simple main effects: ps<0.04 at 13, 14 and 15 weeks for female and 15 weeks for male R6/2 mice). Minocycline did not affect WT mice body weight nor their rotarod (C) performance although the 0.375% dose briefly increased latency to fall in R6/2 mice (treatment×age×day interaction: F(15,294) = 2.5, p<0.003; simple main effects: p<0.05 at 4 weeks of age only during the 3^rd^ day of testing). The 0.375% dose slightly decreased locomotion (D) in the open field in treated R6/2 mice (treatment×age interaction: F(6,179) = 3.96; p<0.002; treatment×age×session time: F(45,895) = 2.28, p<0.0001; simple main effects: ps<0.05 at 6 weeks (0–5 min), 8 weeks (5 and 15–20 min) and 12 weeks (10–15 min)) but did not affect the WT mice. The 0.1% dose of minocycline transiently increased rearing (E) in the R6/2 mice at 4 weeks of age but the 0.375% dose decreased rearing at 6 weeks of age (treatment×age interaction: F(6,179) = 2.9, p<0.015; simple main effects: ps<0.04). The 0.375% dose also decreased rearing in male WT mice at 8 and 12 weeks of age (treatment×age: F(6,186) = 2.4, p<0.03; treatment×gender×age: F(9,186) = 2.15, p<0.03; simple main effects: p<0.05). Neither dose affected grip strength (F) or climbing (G).

In the R6/2 mice, as shown in Fig. 4, we observed a negative effect on survival of both doses of minocycline. Whereas the low dose transiently increased body weight and rearing, the high dose reduced them. The 0.375% dose of minocycline had a minor and transient beneficial effect on rotarod performance, and slightly decreased the locomotor activity in the open field, while no effect was detected in the low dose treated group. Although the 0.375% dose of minocycline also decreased the rearing in male WT mice, it had no effect on any other measure (see legend of figure 4 for statistical details).

Table 2 shows minocycline concentrations in plasma and brain after 44 days of treatment. In contrast to what was found in the 5 mg/kg i.p. dosing study, brain and plasma concentrations of minocycline in R6/2 mice were significantly higher than in WT mice treated with 0.375% of minocycline. Overall brain concentrations, however, were lower than those reported by Smith et al.: 14.5 µM (WT) and 15.5 µM (R6/2), at 1 mg/ml (about equivalent to 0.08% in the food); and 18.7 µM (WT) and 20.5 µM (R6/2) at 5 mg/ml (0.375%).

## Discussion

The goal of the present study was to test an optimized husbandry and behavioral test battery for the evaluation of candidate therapeutic compounds, and reassess with this battery, two compounds tested in preclinical and clinical trials. Our results indicate that high doses of CoQ10 did not ameliorate health, motor, or behavioral symptoms or slow down progression in a mouse model of HD. No dose of minocycline produced any beneficial effects while the highest dose had a clear negative effect on survival and other endpoint measures.

We have developed our drug screening assay to ensure that each treatment group was as similar as possible in terms of biological factors like CAG repeats, initial body weight, and litter size, in order to minimize any potential confounding factors. In addition, all these studies included only animals from healthy litters where more than three pups were viable until weaning, to ensure that any deficits observed cannot be attributed to extraneous factors. In addition, in other studies under the same conditions, we did not observe a clear phenotype indicating imminent death in the R6/2 mice. In particular, we have investigated whether widely used criteria like loss of righting reflex or loss of response to tactile stimulation correlated with death within a 24-hr period and could not detect a relationship. Therefore, no surrogate endpoint for euthanasia was available, in contrast to many other published studies, and survival data reflects the animals' death from natural causes.

### CoQ, nutrition and enrichment

Unlike a previous study reporting that HydroQsorb CoQ10 extends survival in R6/2 mice to a greater degree and at a lower concentration than standard CoQ10 formulations [14], we found that neither formulation showed efficacy even though doses were similar to the ones previously used. Important differences between the studies previously published and the results reported here include animal husbandry conditions and criteria of euthanasia (see above).

In the present study, plasma levels of mice receiving CoQ10 were 0.5 to 1.6 µg/ml, which is in line with other studies [19], [39]. The brain levels we measured after treatment with CoQ10, 13.2–14.2 µg/g, are also in line with other studies [10].

Differences in intake, drug metabolism and/or fat content might have also impacted CoQ10 plasma levels achieved in R6/2 and WT mice. Levels of brain CoQ, which in humans is predominantly CoQ10 and in rodents CoQ9, are affected by nutrition [40], thus the concentrations we observed in the brain may reflect the physiologically normal level of the compound when nutrition is adequate. If the level falls below this concentration, dietary supplements can be used to increase it, as achieved in Smith et al's study who found decreased basal levels in brain CoQ10 in R6/2 mice as compared to WT controls [14]. Indeed, it has been suggested that it is only possible to increase CoQ10 levels in brain when there is a prior deficiency [41]. Consistently, although unexpectedly, the concentrations of CoQ measured in this study did not show a deficiency in the untreated R6/2 mice.

We designed our drug screening system to reduce non-specific health problems, such as suboptimal nutrition due to progressively reduced access to food as the motor dysfunction becomes critical serious [29]. These health issues are secondary to the core HD symptoms and not particularly enticing as functional endpoints for drug discovery. For example, we used lowered waterspouts and delivered food on the floor to enhance nutrient access and thereby reduce the identification of compounds that non-specifically ameliorate malnutrition. These measures are likely to help ensure appropriate nutrition of all mice, including those with motor deficits. On the other hand, HD pathology has been shown to be present even under a well-controlled diet [42] and maybe due to hypothalamic dysfunction [43] and not to nutritional factors, and therefore optimized nutrition, as used in this study could better mimic the human situation.

In this study, animals were housed in an enriched housing environment with the addition of play tunnels, shredded paper and a plastic bone to each cage. Standard housing conditions in US labs typically lack the range of stimuli rodents are normally exposed to in their natural habitats [44]. It is possible that environmental enrichment may confound mouse trials by increasing the heterogeneity of the clinical and neuropathological phenotype [14]. However, if shown empirically that enrichment increases heterogeneity, studies can then be powered appropriately, as done in the studies reported here.

The more relevant question is therefore, whether studying mice in a standard, non-enriched generates false positives by augmenting the chance of therapeutic effects secondary to HD pathology. Housing conditions alone improve motor performance and weight retention in several mouse models of HD [17], [45], [46] affecting the levels of several brain proteins [47], brain volume [45], and hippocampal neurogenesis [48]. For example, Schilling and colleagues have consistently showed a beneficial effect of enrichment in the HD-N171-82Q, which is comparable to that obtained with CoQ at 11 weeks of age [17]. Although those experiments were not designed with this comparison as a goal, this view is consistent with the suggestion that enriched husbandry practices alone may improve energy balance in such a way that putative beneficial treatment effects (e.g. due to CoQ10) are no longer apparent.

In humans, CoQ10 was well tolerated and safe at doses up to 3 g/day [49]. A randomized placebo-controlled, double-blind trial with a dose of 600 mg/day showed a statistically non-significant trend toward slowing in total functional decline with some secondary measures showing positive trends. i.e. functional assessment checklist and few cognitive measures [50]. A phase III clinical trial is currently ongoing to test efficacy of 2.4 g/day (http://clinicaltrials.gov/; study NCT00608881). In addition, CoQ10 analogues that can achieve higher levels of the drug in brain should be evaluated in HD. Along those lines, EPI-743, a bioisostere of CoQ10, was found to have beneficial effects in a small clinical trial for Leigh Syndrome (presented by Guy Miller, in the 5h Annual CHDI Therapeutic Conference, 2010).

### Minocycline, dosage and toxicity

In our evaluation of minocycline, we observed that the low dose (5 mg/kg i.p.) showed some transient, statistically significant effects in drug treated R6/2 mice on body weight, locomotion and rearing, consistent with Chen et al's study, although the effects were not comparable to those published in effect size and duration [25]. In addition, we found that high doses of minocycline resulted in toxic effects with no amelioration of the R6/2 phenotype, consistent with Smith et al's findings [26]. Our results extend the published studies as we used an expanded behavioral testing battery that includes measures of survival not available in Smith et al. study because of animal-welfare regulations at King's College, London, UK.

Smith et al. studied huntingtin aggregation in an organotypic culture and found that minocycline at 30 µM inhibited aggregate formation. In the R6/2 mouse model, however, doses required to achieve similar concentrations in the brain were toxic, therefore lower doses were used (1 and 5 mg/ml in the drinking water) for assessment, which resulted in a concentration of 12.2 µM of minocycline in the R6/2 brain after 5 days of treatment, very similar to the results in our study. Using this maximum tolerated dose, Smith et al. showed neither significant behavioral benefits nor a reduction of huntingtin aggregation [26]. Hersch and colleagues [51] point out three main reasons why Smith et al. may have failed to reproduce Chen et al's positive findings. First, they note that Smith et al.'s initial use of a high dose of minocycline (10 mg/ml), which was later reduced to 5 mg/ml during the trial due to toxicity, may have resulted in irreversible toxic effects. They also point out that Smith et al.'s oral administration is not comparable to their intraperitoneal injections—and questioned Smith et al's measurement of brain minocycline concentration after a single injection as a valid means of estimating the compound's concentration, whereas Chen and colleagues' study involved repetitive dosing. Finally, Hersch and co-workers raised concerns that Smith et al.'s results may have been compromised by the stability of minocycline solutions, which were prepared weekly rather than daily.

We also administered the 5 mg/kg dose using daily i.p. injection as in Chen at al.'s study, and prepared solutions fresh daily. We also ran all behavioral assays at least 3 hours after administration of the injections to avoid acute effects associated with transient high doses of the drug in plasma [38] and measure concentration after chronic dosing. In our study, where minocycline was formulated in the food, the preparation was made daily to minimize problems due to compound stability.

There are, however, other key differences between the two reported studies that could explain the different outcomes. Differences in husbandry conditions and the particular type of R6/2 mice (CAG repeats, in particular) could account for the differing results. Smith et al. bred the R6/2 mice in-house, in an enriched environment, and provided mice with wet mush food, whereas Chen et al. received mice from Jackson Laboratories and then housed the mice under non-enriched conditions without supplementation with wet mush food [25], [26]. Although the exact CAG repeat numbers for the published studies are not published, they are probably close to the 150 CAG reported for mice in Stack et al. study and deposited at Jackson Laboratories by Dr. Bates (Stack et al, Bates personal communication), and thus higher than the numbers measured for the mice in this studies (∼110–125). Possibly, due to a combination of one of these factors but also due to different ways to assess survival, the average lifetime reported of R6/2 mice differed greatly to a point that the study results may not be comparable. Chen et al. reported an average survival of 86 days. Whereas, Smith et al. did not determine average lifetimes, but in the studies reported here, using essentially the same enriched husbandry conditions, we found that untreated R6/2 mice had an average lifetime of 105±8 days (males) and 123±7 days (females), in one cohort, and 109±10 days (females) and 113±7 days (males) in a second cohort. There were also clear differences in the motor testing protocols. Chen et al. used a constant speed (5 or 15 rpm) rotarod protocol whereas Smith et al. (as in our study) used an accelerating rotarod, which may be a more sensitive indicator of progression of disease motoric dysfunction in the R6/2 mouse [52].

To determine if doses of minocycline higher that that used by Chen et al. might be therapeutic we tested doses in the range as reported by Smith et al. [26] but found them to be toxic, consistently with several papers that reported minocycline toxicity in R6/2 [26], the 3-NP model [28], in two models of PD [28], [53] and in a model of hypoxic–ischemia [54]. The negative effects of minocycline have been potentially linked to its inhibitory effects on angiogenesis [55], or to it potential upregulation of prostaglandin 2 and COX-2 production [56], which could result in neuronal injury rather than neuroprotection, although others have shown reduction of such factors instead of upregulation [57].

In the R6/2 studies, we report here, the wild-type controls did not show negative effects of minocycline, apart from decreasing locomotor activity, suggesting that disease pathology may increase sensitivity to possible side effects. Studies in humans have shown little or no toxicity in the doses tested although minor to significant toxic side effects have also been observed in humans including lupus erythematosus-like syndrome, hepatitis, vestibular disturbance, candida infection, gastrointestinal disturbance, urticaria and benign intracranial hypertension [58], [59], [60], [61], [62], [63], [64], [65], [66], [67]. Importantly, in two studies with ALS patients taking high doses of minocycline -of up to 400 mg/day- observations of elevated hepatic enzymes, faster deterioration in the ALS functional measures, and a trend towards a faster decline in forced vital capacity and higher mortality have been reported [68], [69].

In HD patients, minocycline was studied in four clinical trials. A small open-label trial of 100 mg/day showed improvement of general motor and mini mental status exam measures after 6 months and amelioration of psychiatric symptoms and stabilization of motor and neuropsychological function after 24 months [70], [71]. Two other studies showed that 200 mg/day of minocycline was well-tolerated, with no change in HD symptoms [72] although the second study described negative effects in platelet count and an increase in urea. Finally, a double-blind multicenter study, the DOMINO study, did not show a meaningful slowing of the rate of functional decline (http://www.movementdisorders.org/).

### Standardization in preclinical studies

Several factors may contribute to the discrepancies among the several preclinical studies. Colonies of R6/2 mice have been established around the world, arising from two founder lines from the Bates lab (Bates, personal communication). Despite their common origin, the instability of the CAG repeats resulted in drifts in CAG length in individual R6/2 colonies worldwide. It is clear now that such differences account for a change in the onset and severity of disease in the R6/2 mouse [73], [74]. Thus, even purchasing animals from major vendors may result in cohorts with varying repeat lengths than those specified by the original vendor, especially if colonies are established in the investigators' labs and breeders are not selected for a target standard CAG repeat number. Indeed, major steps have been taken in the last few years toward better control of CAG stability with the implementation of high-resolution CAG sizing of Jackson Labs' R6/2 colonies. In addition, the CAG lengths for individual mice in preclinical studies need to be measured and matched prior to treatment assignment, to reduce rates of false positive or negative results, as there may be imbalances of CAG sizes represented across drug and vehicle control groups

In addition, the plasma levels of CoQ10 (0.6%) and brain and plasma minocycline in R6/2 mice (0.375% dose) were higher than in WT mice suggesting either higher food intake or differences in PK. However, when 5 mg/kg minocycline was administered i.p., concentrations were lower, instead, in R6/2 mice. In one study, food intake was slightly increased in young R6/2 mice, consistently with increased our observed drug concentrations, although it was decreased in older mice when clear motor deficits became evident [75]. Thus, an explanation for the observed differences in drug concentration needs further studies.

We also included large numbers of animals to meet minimal power criterion as well as more behavioral assays, resulting in comprehensive assessment. In addition to the commonly used rotarod assay, we include measurements of distance traveled in the open field, rearing and climbing behaviors. Our survival measurements were not based on surrogate measures such a prodding inactive mice [13], [14], [15], [25], and may have resulted in a more direct assessment of lifespan.

In addition to the sources of variability discussed above, biases in the prediction of treatment efficacies are likely to exist due to the overrepresentation of small studies reporting beneficial results. Plots of the relationship between study quality (1/variance of reported results) and effect size in the literature on amyotrophic lateral sclerosis, for example, suggest that small, high variance studies that yield positive results are common in the literature, whereas those that yield negative results are nearly absent [76]. This type of bias is likely to pervade the health-related literature, including that covering HD [77]. We suggest, as have others, that there is a need to create a forum for the publication of preclinical results, including negative studies, which will help view all evidence leading to clinical trials [78], [79].

With the preclinical and clinical evidence at hand, given the safety profile of CoQ, the major problem associated with its use seem to be of economic, given the high cost to the patient of the available CoQ formulations and the expense of large clinical trials powered for small treatment effects [50]. For minocycline, given the frequency of adverse reactions in the healthy population and the lack of robust therapeutic effects, it seems unwarranted to continue testing normal doses (∼200 mg/day) and unsafe to use higher doses (∼400 mg/kg) in patient populations.
